# Validity and reliability of the Turkish version of the 8-item jaw functional limitation scale

**DOI:** 10.22514/jofph.2025.035

**Published:** 2025-06-12

**Authors:** Nazım Tolgahan Yıldız, Hikmet Kocaman, Hasan Bingöl

**Affiliations:** ^1^Department of Physiotherapy and Rehabilitation, Faculty of Health Sciences, Karamanoğlu Mehmetbey University, 70100 Karaman, Turkey; ^2^Department of Physiotherapy and Rehabilitation, Faculty of Physical Therapy and Rehabilitation, Bingöl University, 12000 Bingöl, Turkey

**Keywords:** Functional limitation, Masticatory system, Psychometric, Reliability, Validity, Temporomandibular disorder, Questionnaire

## Abstract

**Background**: Considering that temporomandibular disorder (TMD) can negatively affect individuals’ general health and quality of life by limiting orofacial functions, it is important to evaluate jaw-related functional limitations in TMD. This study aimed to investigate the psychometric properties of the Turkish version of the 8-item Jaw Functional Limitation Scale (JFLS-8-TR) in Turkish-speaking TMD patients. **Methods**: A total of 162 patients with TMD and 114 individuals without TMD participated in the study. Face and content validity were assessed. To confirm the construct validity of the JFLS-8-TR, structural (with confirmatory factor analysis (CFA)), convergent (with pain severity and maximum mouth opening (MMO), Oral Health Impact Profile (OHIP-14), Fonseca Anamnestic Index (FAI) and the Turkish version of the 20-item JFLS (JFLS-20-TR)) and known-group validity were analyzed. Cronbach’s alpha and intraclass correlation coefficient (ICC) were calculated to examine internal consistency and test-retest reliability. **Results**: Both content and face validity were met. The acceptable model fit indices based on CFA confirmed the structural validity of the JFLS-8-TR. Robust associations between the JFLS-8-TR score and pain intensity (*r* = 0.92), MMO (*r* = −0.87) and scores on the FAI (*r* = 0.89), OHIP-14 (*r* = 0.86) and JFLS-20-TR (*r* = 0.96) supported the convergent validity (*p* < 0.05). The JFLS-8-TR was able to discriminate between individuals with and without TMD, indicating that it has known-group validity (*p* < 0.05). The internal consistency (Cronbach’s alpha = 0.91) and test-retest reliability (ICC = 0.93) of the JFLS-8-TR were excellent. **Conclusions**: The JFLS-8-TR is a valid, reliable and useful instrument for a more practical assessment of functional limitations in the masticatory system in Turkish-speaking TMD patients.

## 1. Introduction

Temporomandibular disorders (TMDs) are the second most common musculoskeletal condition affecting the masticatory muscles, temporomandibular joints (TMJs) and adjacent structures, causing pain and disability and affecting a significant proportion of the population [1]. The most prevalent symptoms of TMD are pain in the TMJ or masticatory muscles, limited jaw movement, difficulty in chewing, clicking sounds and headache [2, 3]. These symptoms reduce individuals’ quality of life by interfering with basic activities of daily living such as chewing, smiling, speaking, eating and social participation [1, 2]. TMD has a multifactorial etiology with biological, behavioural and psychosocial factors [3]. A recent study reported that the overall prevalence of TMD is 31% in adults and the elderly and 11% in adolescents and children [4]. Research has shown that TMD is significantly common in the Turkish community, with prevalence ranging from 22.4% to 41.8% [2, 5].

Orofacial function contributes significantly to a person’s overall health and quality of life. However, orofacial disorders such as TMD negatively impact an individual’s quality of life by limiting the orofacial and psychosocial function [6, 7]. It is important to assess jaw-related functional impairment in addition to symptoms in individuals with TMD, as TMD can impair oral-facial function [1, 2, 8]. In orofacial disorders, specific instruments have been designed to evaluate the functional limitations in the jaw [1]. Among these instruments, the Jaw Functional Limitation Scale (JFLS), which is available in 20-item and 8-item versions was developed to assess the functional limitation of the masticatory system [6]. The use of this instrument in research and clinical settings for patient populations with functional limitations of the masticatory system has been suggested by both the Diagnostic Criteria for TMD (DC/TMD) [1] and the literature [2, 6, 8, 9, 10, 11]. The JFLS was originally developed as an 8-item instrument (JFLS-8) [9]. Subsequently, a 20-item version (JFLS-20) with three subscales and a global scale was developed based on this 8-item version [6]. The JFLS-20 has been shown to be reliable and valid for use in TMD patients in its original form [6], as well as in Malaysian English [10], Croatian [11], Chinese [8] and Turkish [2] adaptations. The JFLS-8, which is more concise than the JFLS-20 and more practical for use in busy clinical settings, has been validated and shown to be reliable in its original form [9]. The JFLS-8 has been identified as an appropriate tool for efficiently assessing functional limitations in the masticatory system [9]. Its creators have indicated that this practical instrument has excellent validity, sensitivity to change and reliability [6, 9].

Assessment of the functional limitation of the masticatory system in TMD utilizing the JFLS-8 can provide valuable insights for identifying patient needs, selecting appropriate interventions and monitoring treatment-related improvements. For improved practicality in busy clinical settings, the JFLS-8 may be preferred over the JFLS-20, as it allows for evaluation of a greater number of patients in a shorter period of time [1, 6]. Although the JFLS-8 was previously translated into Turkish by Polat *et al*. [12] in 2016, the validity and reliability of the Turkish version of the JFLS-8 (JFLS-8-TR) in patients with TMD have not been previously established. Considering the high prevalence of TMD, and the substantial patient volume in dental clinics, the need for the JFLS-8 to more efficiently assess jaw functional limitations in TMD patients is evident. Therefore, this study aimed to investigate the psychometric properties of the JFLS-8-TR in Turkish-speaking TMD patients.

## 2. Materials and methods

### 2.1 Study design and ethical aspects 

This observational and cross-sectional research was approved by the Ethics Committee for Scientific Research and Publication of Muş Alparslan University (number: 09-2023/56). Study data were collected from patients with TMD and healthy individuals without TMD between December 2023 and September 2024. Written informed consent was obtained from all participants who were informed about the study. The entire procedure was conducted according to the tenets of the Declaration of Helsinki. The following aspects of measurement characteristics were assessed and reported according to the COSMIN (Consensus Based Standards for the Selection of Health Measurement Instruments) taxonomy [13].

### 2.2 Instrument

The JFLS-8 was originally developed by Ohrbach *et al*. [9] as an 8-item organ-specific instrument to assess functional limitation of the masticatory system in orofacial conditions such as TMD. The instrument measures the degree of limitation in eight functional activities using an 11-item numerical rating: chewing chicken (*e.g.*, baked), chewing hard food, eating soft food that does not require chewing (pureed food, pudding), swallowing, opening wide enough to drink from a cup, yawning, smiling and talking. Each item is answered on a scale from 0 (no limitation) to 10 (severe limitation). The total score from the eight items is averaged to give a global limitation score ranging from 0 to 10. A higher global score indicates a more severe limitation related to the jaw [6, 9]. The JFLS-8 was previously translated from English into Turkish by Polat *et al*. [12] in 2016. The Turkish version of the JFLS-8 (JFLS-8-TR) is available at: https://inform-iadr.com/index.php/tmd-assessmentdiagnosis/dc-tmd-translations/. Although the JFLS-8 has been translated into Turkish by Polat *et al*. [12], the validity and reliability of the scale in Turkish-speaking patients with TMD have not been confirmed. Therefore, in this study, the JFLS-8-TR was used to investigate its validity and reliability in Turkish-speaking TMD patients after obtaining permission from the researchers.

### 2.3 Participants

The study enrolled 162 patients diagnosed with TMD in private dental clinics in Karaman province and Karamanoğlu Mehmetbey University Faculty of Dentistry and 114 healthy individuals who were confirmed to not have TMD in Karamanoğlu Mehmetbey University Faculty of Dentistry. To explore the known-group validity of the JFLS-8-TR, 114 healthy individuals were recruited who had comparable demographic properties (age, gender, body mass index) to the patients, were literate in Turkish, were 18 years of age or older, and were confirmed without TMD through undergoing clinical examination by specialist dentists with at least 10 years of experience in field of TMD. Of the 162 TMD patients, 54 patients were included from two private dental clinics in Karaman province, and 108 patients were included from Karamanoğlu Mehmetbey University Faculty of Dentistry. Patients were examined clinically and radiologically by specialist dentists with at least 10 years of experience in the field of TMD, and then diagnosed with TMD using the Diagnostic Criteria for TMD (DC/TMD) Axis I [1]. For the TMD patients, the inclusion criteria were as follows: having TMD complaints for at least six months and being diagnosed with TMD, being over 18 years of age, being literate in Turkish, not receiving any actual treatment for the orofacial region, and TMD such as splinting or analgesic medication. Patients with TMD complaints of at least six months’ duration were included in this study for the following reason: previous research has established a strong association between chronic TMD and functional limitations of the jaw. In addition, it has been reported that significant TMD-related functional limitations typically develop approximately six months after the onset of the disorder (chronic TMD) [2]. Exclusion criteria for all participants were: a history of trauma or surgery to the neck, head or TMJ; diagnosed systemic and psychiatric diseases; diseases that may affect the TMJ, such as ankylosing spondylitis or rheumatoid arthritis; and pregnancy. The flow diagram of the study participants was illustrated in Fig. 1.

**Fig. 1.** The flow diagram of the study participants. TMD: Temporomandibular disorder.

As there is no set method for quantifying sample size in psychometric research, the appropriate sample size for the present study was calculated using a number of principles. First, the patient-item ratio, which suggests 5–10 participants per item, was considered in the sample size calculation [14]. Secondly, we considered the sample size calculation, which suggests 10–20 participants for each variable for factor analysis [15]. According to the above hypotheses, a sample of over 80 patients with TMD was considered adequate, given that the JFLS-8-TR consists of eight items.

### 2.4 Data collection procedure

To assess the known-group validity of the JFLS-8-TR, healthy individuals without TMD were instructed to complete only the JFLS-8-TR without any additional assessments. Meanwhile, patients with TMD completed all of the following assessments.

Patients’ pain intensity during jaw activity was measured using a visual analogue scale (VAS). Patients labelled the intensity of pain they felt on a 10 cm line, with “no pain” at one end and “most severe pain” at the other. Pain intensity was recorded in cm (VAS cm). For the measurement of active painless maximum mouth opening (MMO), the patient was asked to open the mouth as wide as possible without feeling any pain. The distance between the upper and lower central incisors was measured with a ruler and the value was registered in millimetres [5, 16]. A Turkish version of the Fonseca Anamnestic Index (FAI), which has been shown to be valid and reliable [17], was used to assess the severity of TMD [2]. The FAI is one of the most commonly used screening tools to detect both the presence and severity of TMD [18]. The presence and severity of TMD are categorized as follows, based on the total score obtained by summing the responses to the 10 questions in the scale: 0–15 (no TMD), 20–40 (mild TMD), 45–65 (moderate TMD) and 70–100 (severe TMD) [17]. In the instrument, where the total score ranges from 0 to 100, higher scores can be interpreted as greater TMD severity, as noted in previous studies [2, 19]. In the previous study [2], the convergent validity of the Turkish version of the 20-item Jaw Functional Limitation Scale (JFLS-20-TR) was confirmed based on the strong relationships between the JFLS-20-TR scores and the FAI score. Accordingly, in the present study, to investigate the convergent validity of the JFLS-8-TR, the relationship between the JFLS-8-TR score and the FAI score was assessed. The Turkish version of the 14-item Oral Health Impact Profile (OHIP-14), with established validity and reliability, was administered to assess oral health-related quality of life. The instrument has an overall score ranging from 0 to 56, with a higher score indicating poorer oral health-related quality of life [20]. The JFLS-20-TR was administered to assess functional limitation in the masticatory system [2]. The scale has three sub-domains (vertical jaw mobility, mastication and verbal and emotional expression) and 20 items, with responses to each item ranging from 0 (no limitation) to 10 (severe limitation). The overall score, which ranges from 0 to 10, is calculated by averaging the responses to the items. A high overall score reflects greater functional limitation [2, 6].

### 2.5 Validity

#### 2.5.1 Content and face validity

Twenty specialist dentists with at least 10 years of experience in the field of TMD evaluated the JFLS-8-TR for content validity. After reviewing the items in the JFLS-8-TR, the experts categorized each item as “essential”, “useful but not essential” and “not essential”. To test the content validity of the scale, the content validity index (CVI) was calculated as in previous studies [5, 21]. To assess the face validity of the JFLS-8-TR, 28 TMD patients were instructed to complete the scale and rate the items in the scale as “incomprehensible”, “a little confusing” and “comprehensible”. A comprehension index, corresponding to the proportion of “comprehensible” responses, was then calculated for each item [5]. For content and face validity, CVI and comprehension index values equal to or greater than 0.80 and 0.83, respectively, were considered satisfactory [22].

#### 2.5.2 Construct validity

To assess the construct validity of the JFLS-8-TR, structural, convergent and known group validities were analyzed [23]. Confirmatory factor analysis (CFA) was used to test the structural validity of the JFLS-8-TR. The model fit indices of the CFA were accepted as adequate for model fit if they were within the following ranges: comparative fit index (CFI) >0.90, adjusted goodness of fit index (AGFI) >0.90, ratio of the chi-square statistic to degrees of freedom (χ^2^/*df*) <5, standardized root mean square residual (SRMR) 0 < α < 0.05, root mean square error of approximation (RMSEA) <0.080 and goodness of fit index (GFI) >0.90 [5, 24]. To investigate convergent validity, potential associations between the JFLS-8-TR score and pain intensity, MMO, and scores on the FAI, OHIP-14, and JFLS-20-TR scales were assessed. A correlation coefficient greater than 0.60 was considered as strong, between 0.30 and 0.60 as moderate, and less than 0.30 as low correlation [5]. One method of confirming the construct validity of a questionnaire is to investigate its known-group validity through hypothesis testing. In this study, to assess the known-group validity of the JFLS-8-TR, in other words, to establish whether the instrument can discriminate between TMD patients and individuals without TMD, the scores of patients and of 114 individuals without TMD on the JFLS-8-TR were compared [5, 23].

### 2.6 Reliability

#### 2.6.1 Internal consistency

Cronbach’s alpha coefficient was calculated to analyse the internal consistency of the JFLS-8-TR. An alpha coefficient greater than 0.70 was considered acceptable, while a coefficient equal to or greater than 0.80 was considered to indicate excellent internal consistency [5, 25].

#### 2.6.2 Test-retest reliability

To assess the test-retest reliability of the JFLS-8-TR, retest patients were randomly selected by assigning each patient a unique identifier in a Microsoft Excel (version 2016, Microsoft Corp., Redmond, WA, USA) RANDBETWEEN function (*e.g.*, 1 to 162). Of 162 TMD patients who had previously completed the scale, 134 were randomly selected for the retest group using the RANDBETWEEN function. These 134 patients were contacted one day before the retest assessment, and 24 patients whose pain intensity changed by more than one unit on the VAS were excluded from the retest group. As a result, 110 randomly selected TMD patients whose pain intensity did not change more than one unit on the VAS [2, 26] and whose clinical status was stable completed the JFLS-8-TR again after seven days. To assess test-retest reliability, the intraclass correlation coefficient (ICC) was calculated. ICC values less than 0.5 were considered as poor reliability, between 0.5 and 0.75 as moderate reliability, between 0.75 and 0.9 as good reliability, and greater than 0.90 as excellent reliability [27].

### 2.7 Feasibility

To assess the applicability of the JFLS-8-TR, the completion time of the questionnaire was recorded in minutes for all TMD patients.

### 2.8 Statistical analysis

Except for the CFA, statistical analyses were performed using IBM SPSS Statistics (version 25.0, IBM Corp., Armonk, NY, USA). The CFA was conducted using the statistical software IBM SPSS AMOS (version 20.0, IBM Corp., Armonk, NY, USA) statistical software. Descriptive statistics for continuous variables were presented as mean ± standard deviation (M ± SD), while categorical variables were given as percentages or frequencies. Visual tests (histogram and normal quantile-quantile plot) and the Kolmogorov-Smirnov test were used to determine whether numerical variables were normally distributed. An independent samples *t*-test was used to compare age and body mass index between TMD patients and individuals without TMD, and a Chi-squared test was used to compare gender distribution. CFA was used to assess whether the previously defined single-factor structure of the JFLS-8-TR was adequately supported by the collected data from Turkish-speaking TMD patients. Thus, the structural validity of the scale was analyzed [5, 24]. To determine the convergent validity of the scale, the associations between the JFLS-8-TR score and pain intensity, MMO, and the scores of the FAI, OHIP-14 and JFLS-20-TR scales were examined using Pearson correlation analysis. As in the previous study [5], the power of the associations was assessed based on the correlation coefficient obtained. Known-group validity was assessed by comparing the scores of TMD patients on the JFLS-8-TR with those of individuals without TMD using an independent-samples *t*-test. Statistical significance was considered to be *p* < 0.05.

## 3. Results

We excluded 45 patients who refused to participate in the study or who did not meet the inclusion criteria out of 207 patients who were referred to us with a diagnosis of TMD. In addition, 23 out of 137 healthy individuals who were referred to us and found not to have TMD were excluded because they refused to participate in the study or did not meet the inclusion criteria. Consequently, the study was completed with 162 patients with TMD and 114 individuals without TMD. Fig. 1 illustrates the flow diagram of the study participants. The clinical and demographic characteristics of the participants are listed in Table 1. Patients with TMD and individuals without TMD had a similar age, body mass index and gender distribution (*p* > 0.05). In addition, the mean scores of TMD patients on the JFLS-8-TR were statistically higher than those of individuals without TMD (*p* = 0.004) (Table 1).

**Table 1.** Demographic and clinical characteristics of the participants.

Variables		Patients with TMD (n = 162)	Individuals without TMD (n = 114)	*p*
Gender				
	Female, n (%)	106 (65.4)	72 (63.2)	0.457^a^
	Male, n (%)	56 (34.6)	42 (36.8)	
Age (yr), M ± SD		35.55 ± 5.61	36.48 ± 6.03	0.329^b^
BMI (kg/m^2^), M ± SD		23.82 ± 2.31	24.37 ± 2.64	0.683^b^
JFLS-8-TR score, M ± SD		4.98 ± 1.76	0.13 ± 0.02	**0.004^b^**
Pain intensity (cm) , M ± SD		5.86 ± 1.82		
MMO (mm) , M ± SD		27.36 ± 6.14		
FAI score, M ± SD		56.27 ± 14.06		
OHIP-14 score, M ± SD		29.72 ± 7.16		
JFLS-20-TR score, M ± SD		4.79 ± 1.64		
TMD diagnostic subgroups		n (%)		
Myofascial pain with referral		40 (24.7)		
Local myalgia		35 (21.6)		
Arthralgia		22 (13.6)		
Disk displacement with reduction		37 (22.8)		
Disk displacement without reduction		17 (10.5)		
Degenerative joint disease (osteoarthritis)		11 (6.8)		

*TMD: Temporomandibular disorder; M ± SD: Mean ± standard deviation; BMI: Body mass index; JFLS-8-TR: Turkish version of the 8-item Jaw Functional Limitation Scale; MMO: Maximum mouth opening; FAI: Fonseca Anamnestic Index; OHIP-14: 14-item Oral Health Impact Profile; JFLS-20-TR: Turkish version of the 20-item Jaw Functional Limitation Scale; p^a^: Chi square test, p^b^: Independent sample t-test; bold format indicates p < 0.05.*

### 3.1 Face and content validity

In terms of the content validity of the JFLS-8-TR, the CVI scores for each item in the instrument ranged from 0.90 to 1.00, while in terms of face validity, the comprehension index scores for each item ranged from 0.89 to 1.00 (Table 2).

**Table 2.** Content and face validity of the JFLS-8-TR.

	Content validity			Face validity		
Item	Essential (n)	Useful but not essential (n)	CVI	Comprehensible (n)	A little confusing (n)	Comprehension index
1	20	-	1.00	26	2	0.93
2	19	1	0.95	26	2	0.93
3	18	2	0.90	25	3	0.89
4	19	1	0.95	26	2	0.93
5	18	2	0.90	25	3	0.89
6	18	2	0.90	28	-	1.00
7	19	1	0.95	27	1	0.96
8	18	2	0.90	26	2	0.93

*JFLS-8-TR: Turkish version of the 8-item Jaw Functional Limitation Scale; CVI: Content validity index.*

### 3.2 Construct validity

In terms of the structural validity of the JFLS-8-TR, the standardized factor loadings of each item in the scale were above 0.70, which is an acceptable value varying between 0.83 and 0.94, indicating that all items in the JFLS-8-TR explained the latent variable sufficiently and no item needed to be excluded from the scale. Furthermore, the model fit indices obtained from the CFA were within acceptable limits, which confirmed the structural validity of the JFLS-8-TR (Table 3).

**Table 3.** Results of confirmatory factor analysis and model fit indices for the JFLS-8-TR.

		Model fit indices		
JFLS-8-TR Items	Items’ factor loading	Indices	Acceptable values	Model value
Item 1	0.86	χ^2^/*df*	<5.00	3.142
Item 2	0.93	CFI	>0.90	0.941
Item 3	0.92	AGFI	>0.90	0.963
Item 4	0.90	GFI	>0.90	0.932
Item 5	0.94	SRMR	0 < α < 0.05	0.014
Item 6	0.83	RMSEA	<0.08	0.047
Item 7	0.92			
Item 8	0.93			

*JFLS-8-TR: Turkish version of the 8-item Jaw Functional Limitation Scale; χ^2^/df: The ratio of the chi-square statistic to degrees of freedom; CFI; Comparative fit index; AGFI: Adjusted goodness of fit index; GFI: Goodness of fit index; SRMR: Standardized root mean squared residual; RMSEA: Root mean square error of approximation.*

The results of the convergent and known-group validities for the JFLS-8-TR are presented in Table 4. There were strong positive associations between the JFLS-8-TR score and pain intensity and scores on the FAI, OHIP-14 and JFLS-20-TR scales, whereas there were strong negative associations with MMO (*p* < 0.05). Patients with TMD had a significantly higher JFLS-8-TR score when compared to those without TMD (*p* < 0.05) (Table 4).

**Table 4.** Results of convergent and known-group validity and reliability analyses for JFLS-8-TR.

		JFLS-8-TR score	*p*
Convergent validity			
	Pain intensity	*r* = 0.92	**0.012^a^**
	MMO	*r* = −0.87	**0.006^a^**
	FAI score	*r* = 0.89	**0.014^a^**
	OHIP-14 score	*r* = 0.86	**0.019^a^**
	JFLS-20-TR score	*r* = 0.96	**0.003^a^**
Known-group validity			
	Patients with TMD (n = 162), M ± SD	4.98 ± 1.76	**0.004^b^**
	Individuals without TMD (n = 114), M ± SD	0.13 ± 0.02	
Internal consistency	Cronbach’s alpha	0.91	
Test-retest reliability	ICC (95% CI)	0.93 (0.91–0.95)	

*JFLS-8-TR: Turkish version of the 8-item Jaw Functional Limitation Scale; MMO: Maximum mouth opening; FAI: Fonseca Anamnestic Index; OHIP-14: 14-item Oral Health Impact Profile; JFLS-20-TR: Turkish version of the 20-item Jaw Functional Limitation Scale; TMD: Temporomandibular disorder; M ± SD: Mean ± standard deviation; ICC: Intraclass correlation coefficient; CI: Confidence interval; r: Pearson’s correlation coefficient; p^a^: Pearson’s correlation analysis; p^b^: Independent sample t-test; bold format indicates p < 0.05.*

### 3.3 Internal consistency

The calculated total Cronbach’s alpha coefficient for the JFLS-8-TR indicated that the scale had high internal consistency (Table 4). In terms of corrected item-total correlations, the correlation coefficient of each item with the JFLS-8-TR varied between 0.74 and 0.87, confirming that there was a robust association between each item and the JFLS-8-TR total score.

### 3.4 Test-retest reliability

The ICC value calculated for the JFLS-8-TR confirmed that the scale has excellent test-retest reliability and temporal stability (Table 4).

### 3.5 Feasibility

The mean time to complete the JFLS-8-TR for all patients with TMD was 2.31 ± 0.36 minutes (min: 1.9–max: 3.3).

## 4. Discussion

The results of the current research confirmed that the JFLS-8-TR is a feasible instrument with good face, content and construct validity (as structural, convergent and known-group validity), as well as excellent test-retest reliability and internal consistency. Therefore, the JFLS-8-TR is practical, valid and reliable for the assessment of functional limitations in the masticatory system in the Turkish-speaking TMD patients.

In temporomandibular disorders (TMD), which are common in society, functional limitations that adversely affect the masticatory system and cause deterioration in patients’ psychosocial and orofacial functions have a notable impact on the reduction of quality of life. In this respect, in addition to complaints such as pain in TMD patients, the evaluation of functional limitation of the masticatory system is crucial for the identification of needs and the effective management of TMD [1, 2, 6]. Of the tools available to assess jaw-related functional limitations, the JFLS is a valuable organ-specific scale that is recommended by the DC/TMD and is widely used in clinical and research settings [1, 2, 6, 8, 10, 11]. There are the original English [6] and Croatian [11] versions of the JFLS-8, which are shorter than the 20-item version and allow for more practical administration in clinics with high patient volume, both of which have been shown to be valid and reliable.

In the present study, the comprehension index (between 0.89 and 1.00) and CVI (between 0.90 and 1.00) values obtained to examine the face and content validity of the JFLS-8-TR were above the threshold values. Based on these findings, it was concluded that the JFLS-8-TR, which has satisfactory face validity, is appropriate in terms of the feature being assessed and the target population to whom it is administered appears to adequately evaluate the trait it is intended to measure, and each item in the scale is understandable. In addition, the JFLS-8-TR has sufficient content validity, *i.e.*, the extent to which all the items in the instrument accurately reflect/cover the structure/feature being assessed. Although the face and content validity of the JFLS-8 have not been examined in the Croatian version [11], the original 8- and 20-item versions of the scale have been found to have sufficient content validity and to be understandable by patients [6].

We investigated the construct validity of the JFLS-8-TR by analyzing its structural, convergent and known-group validity. Based on the CFA results, the model fit indices were within acceptable ranges, and the standardized factor loadings of each item were greater than 0.70 (ranging from 0.83 to 0.94), supporting that the JFLS-8-TR has satisfactory structural validity. A preliminary version of the JFLS-8 [9] was developed using Rasch and factor analysis, and it was emphasized that the scale showed a good model fit. In the present study, robust relationships between the JFLS-8-TR score and the pain intensity, MMO, and scores on the FAI, OHIP-14 and JFLS-20-TR scales supported the convergent validity of the JFLS-8-TR. These findings suggest that increased pain intensity, mouth opening limitation and TMD severity may worsen jaw-related functional limitations, resulting in a decrease in patients’ quality of life. There is some research in the literature supporting the associations between the JFLS score and pain intensity, TMD severity, and quality of life [2, 8, 9, 11, 28]. While the developers of the JFLS-8 found a moderate relationship between the JFLS-8 score and pain intensity [9], a moderate relationship was also found between the global score of the Chinese version of the JFLS-20 and pain intensity [8]. Another study found that jaw-related functional limitations were primarily related to sleep disturbance, pain intensity and kinesiophobia [28]. The construct validity of the preliminary version of the JFLS-8 was established by analyzing convergent and discriminant validity using specific variables. Based on correlations between the JFLS-8 and the variables somatization, pain-free opening, anxiety, palpation sensitivity, depression, jaw symptoms and pain, the preliminary version was found to have strong construct validity [9]. Research investigating the psychometric properties of the 20-item and 8-item Croatian versions of the JFLS [11] found that TMD patients with higher pain severity had significantly greater jaw functional limitations and emphasised that pain intensity was an important determinant of jaw functional limitations. Recently, a JFLS-20-TR showed strong associations between the JFLS-20-TR global score and pain severity [2]. In the current study, the strong association of the JFLS-8-TR score with MMO is consistent with the previous studies [2, 8], suggesting that reduced mouth opening may exacerbate jaw-related functional limitations. While the JFLS-20-TR score was strongly associated with mouth opening [2], the Chinese version of the JFLS-20 score was moderately correlated with mouth opening, and it has been documented that MMO is an important factor influencing jaw functional limitation in TMD [8]. In this study, the robust associations between the JFLS-8-TR score and the scores of the OHIP-14 and FAI scales suggest that limitations in jaw function may increase the TMD severity and reduce the patients’ quality of life. Previous studies have supported these findings [2, 10]. The convergent validity of the Malaysian English version of the JFLS-20 was established based on the moderate relationships between the JFLS-20 and the OHIP scores [10]. In another study, the convergent validity of the JFLS-20-TR was supported by the robust associations between JFLS-20-TR scores and OHIP-14 and FAI scores [2]. Another notable finding of the present study was the strong correlation of 0.96 between the JFLS-8-TR score and the JFLS-20-TR total score, which has been previously validated and shown to be reliable [2]. This indicates a very high level of agreement between the two Turkish versions of the JFLS. Similarly, a robust correlation of 0.94 was found between the original 20-item and 8-item versions of the JFLS [6]. In the present study, the fact that the scores of TMD patients on the JFLS-8-TR were significantly higher than the scores of individuals without TMD showed that the JFLS-8-TR could discriminate between individuals with and without TMD in terms of jaw functional limitations; therefore, it had known-group validity. Similar findings have been documented in previous studies. The original study of the JFLS-8 [6] and the Turkish [2] and Malaysian English [10] studies of the JFLS-20 concluded that the 20-item and 8-item JFLSs were able to discriminate between individuals with and without TMD, and thus had known group validity.

The Cronbach’s alpha coefficient for the JFLS-8-TR was 0.91, which supported that the scale had high internal consistency for practice in Turkish-speaking society and that all items in the instrument assessed a similar construct. For the original JFLS-8 and JFLS-20, Cronbach’s alpha coefficients were found to be 0.87 and 0.95, respectively, and both versions were reported to have high internal consistency [6]. Fetai *et al*. [11] found that the Croatian versions of the JFLS-8 and JFLS-20 had high internal consistency with alpha coefficients of 0.81 and 0.93, respectively. In addition, the Chinese [8], Malaysian English [10] and Turkish [2] versions of the JFLS-20 were found to have high internal consistency with alpha coefficients of 0.91, 0.96 and 0.93 respectively. The JFLS-8-TR was found to have excellent test-retest reliability with an ICC value of 0.93. This confirmed the temporal stability of the JFLS-8-TR in repeated assessments in Turkish-speaking TMD patients. Ohrbach *et al*. [6] calculated the concordance correlation coefficients for the original English JFLS-8 and JFLS-20 to be 0.81 and 0.87 respectively, and found that both versions had good test-retest reliability. The test-retest reliability of the Croatian version of the JFLS-8 [11] has not been assessed. Furthermore, the Chinese version of the JFLS-20 [8] was reported to have good test-retest reliability (ICC = 0.87), while the Malaysian English [10] and Turkish [2] versions had excellent test-retest reliability (ICC values of 0.97 and 0.95, respectively). The authors also emphasized the temporal stability of all three versions.

The average completion time for the JFLS-8-TR among all TMD patients was 2.31 minutes. The average completion time for the JFLS-20-TR was 4.43 minutes [2]. Therefore, with a short completion time of approximately two minutes, the JFLS-8-TR could be preferred over the JFLS-20-TR for a more practical use in clinics with a high volume of TMD patients.

There were some limitations to this study. The current cross-sectional study could not analyse the responsiveness of the JFLS-8-TR; also, the specificity and sensitivity of the JFLS-8-TR were not investigated. Secondly, overbite was not considered in the MMO measurement. Thirdly, because the TMD diagnostic subgroups were not homogeneously distributed in the study sample, they were not taken into account in the analyses. Fourthly, this study did not categorize patients according to TMD severity as having mild, moderate or severe TMD. Therefore, in the present study, analyses by TMD severity were not performed, and the extent to which functional limitations of the jaw were affected in different TMD diagnostic subgroups or different TMD severities (mild, moderate, severe) could not be evaluated. In future research, in addition to the responsiveness, specificity and sensitivity of the JFLS-8-TR, investigating the extent to which the jaw functional limitations are affected in different TMD diagnostic subgroups and at different TMD severities may be beneficial for clinical and research practice of the JFLS-8-TR. Finally, the fact that only Turkish-speaking patients with TMD in Karaman province of Turkey were included in this study limits the generalizability of the psychometric properties of the JFLS-8-TR to all patients with TMD in Turkey. Considering that the psychometric properties of the JFLS-8-TR may be sensitive to sociocultural characteristics that may vary in different regions of Turkey, it would be useful to examine the psychometric properties of the JFLS-8-TR in Turkish-speaking patients with TMD in different regions of Turkey in further studies. On the other hand, the strength of the present study is that it revealed that the JFLS-8-TR is a valid and reliable instrument for use in Turkish-speaking TMD patients. Moreover, this study provided valuable insights by analyzing the structural validity of the JFLS-8 through CFA.

## 5. Conclusions

The results of this study confirmed that the JFLS-8-TR is feasible and understandable and has good face, content and construct validity, as well as excellent test-retest reliability and internal consistency. In this respect, the JFLS-8-TR is a valid, reliable and useful instrument for the practical assessment of organ-specific functional limitations in the masticatory system in Turkish-speaking TMD patients. The JFLS-8-TR can be practically used in both research and clinical practice to measure jaw-related functional limitations in individuals with TMD.
